# Public Interest in Stress-Induced Dermatologic Conditions and Psychodermatologic Disorders Before, During, and After the COVID-19 Pandemic: Segmented Regression Analysis

**DOI:** 10.2196/93458

**Published:** 2026-09-30

**Authors:** Marisa N Fat, Hayden C Johnson, Michael Bernas, Cheryl L Hurd, Robert C Smith

**Affiliations:** 1 Department of Medicine, Anne Burnett Marion School of Medicine at Texas Christian University, 1100 W Rosedale St, Fort Worth, TX, 76104, United States, 1 (817) 257-6633; 2 Department of Graduate Education, University of California, Los Angeles, Los Angeles, CA, United States

**Keywords:** psychodermatology, psychological stress, COVID-19 pandemic, Google Trends, skin disorders

## Abstract

**Background:**

Psychological stress is known to exacerbate dermatologic conditions such as acne, eczema, and compulsive skin behaviors. The COVID-19 pandemic introduced a global stressor with widely experienced psychosocial effects and potential impacts on skin health. This study analyzes US public interest in stress-induced dermatologic conditions and psychodermatologic disorders to test for pandemic-associated shifts in 6 search terms.

**Objective:**

This study aimed to assess whether the onset and resolution of the COVID-19 pandemic were associated with changes in Google Search interest in psychodermatologic and stress-related skin conditions in the United States.

**Methods:**

Monthly relative search volume (RSV; 0-100 scale) for 6 terms (“skin picking,” “trichotillomania,” “stress rash,” “eczema flare up,” “dermatillomania,” and “anxiety skin picking”) was obtained from Google Trends for January 2018 through December 2024 (n=84 monthly observations per term); the 6 primary and 4 secondary exploratory terms were extracted in April 2025 and the 5 synonymic terms in July 2026, using the identical query window. A segmented regression model with break points at the World Health Organization’s (WHO) pandemic timeline (March 2020 declaration; May 2023 end of concern) tested for level and slope changes at pandemic onset and post pandemic. Newey-West (heteroskedasticity and autocorrelation consistent) SEs corrected for residual autocorrelation, and joint *F* tests with Bonferroni and Benjamini-Hochberg (false discovery rate [FDR]) corrections assessed whether each period differed from the prior trend. A sensitivity analysis applied the identical model to 5 synonymic terms, each paired with 1 primary term, and to 4 unpaired, secondary, exploratory terms (“acne,” “stress acne,” “stress hives,” and “hair loss from stress”).

**Results:**

A total of 2 of the 6 terms showed a statistically significant pandemic-onset effect after correction: “dermatillomania” (level shift +15.20 RSV points; Bonferroni *P*=.007; FDR *P*=.007) and “anxiety skin picking” (slope change +0.70 RSV points/month; Bonferroni *P*=.02; FDR *P*=.01). “Stress rash,” “skin picking,” and “eczema flare up” reached significance under FDR correction only (*P*=.04, .04, and .03, respectively); “trichotillomania” showed no effect under either correction (FDR *P*=.08). “Anxiety skin picking,” “trichotillomania,” and “stress rash” showed postpandemic reversals robust to both corrections. The synonymic sensitivity analysis showed weaker, inconsistent support: only 1 of 5 pairs (“dermatillomania”/“excoriation disorder”) showed partial agreement under FDR correction, while the remaining 4 diverged, indicating sensitivity to search-term phrasing. The secondary exploratory analysis found no pandemic-onset effects under either correction.

**Conclusions:**

Public search interest in a subset of psychodermatologic conditions, specifically “dermatillomania” and “anxiety skin picking,” showed statistically significant shifts temporally coinciding with COVID-19 pandemic onset. A sensitivity analysis using synonym terms did not consistently reproduce these findings, indicating sensitivity to both analytic choices and search-term phrasing. These results support further research into psychodermatology and illustrate how public search behavior can reflect societal stress, though causal interpretation is unwarranted from search-volume data alone.

## Introduction

Psychodermatology is an emerging field that bridges psychiatry and dermatology, 2 specialties with distinct variations but notable clinical overlap [1]. It focuses on the complex interplay between the mind and skin, where psychological stress can exacerbate dermatologic conditions and skin conditions can lead to emotional distress. Psychological stress is well established as being associated with common skin-related conditions, including acne, psoriasis, urticaria, and eczema [1,2]. This is because the interaction between the skin and the immune system is affected by stress, causing dysregulation of immune cells within the skin barrier and making patients more prone to developing a skin disorder [3]. Although previous studies have detailed the likely relationship between psychological stress and dermatologic diseases, little epidemiological analysis has been performed to examine the number of those affected [3].

The COVID-19 pandemic, caused by SARS-CoV-2, was associated with psychological distress and mental health concerns among large numbers of individuals [4]. Prolonged lockdowns, reduced social interaction, and restricted access to daily routines and indoor and outdoor activities affected individuals’ emotional regulation and overall mental well-being, resulting in a 25% increase in anxiety and depression diagnoses during this time [5]. Despite growing mental health concerns, the link between stress and skin conditions during this period remains underexplored [6]. The COVID-19 pandemic has been established as a possible influence on skin conditions such as acne [7], psoriasis [8], urticaria [9], and trichotillomania [10]; however, the literature is sparse.

This study aimed to evaluate changes in public interest in psychodermatologic conditions in the context of the COVID-19 pandemic in the United States, one of the most impactful public health events in recent history. Applying a segmented regression approach to formally test whether the pandemic period represented a statistically distinguishable shift from pre-existing trends provides insight into the effective use of Google Trends data in this space.

## Methods

### Data Source and Search Terms

A list of 6 search terms was constructed to represent commonly recognized psychodermatological conditions used in both medical and colloquial terminology [1]: “skin picking,” “eczema flare up,” “dermatillomania,” “anxiety skin picking,” “trichotillomania,” and “stress rash.” Using stress-qualified terms for the inflammatory and eczematous categories rather than the bare terms “rash” and “eczema” was a deliberate choice intended to capture searches more specifically attributed to a stress-related presentation. This choice avoids nonspecific causes such as allergic or infectious causes that bare dermatologic terms would capture.

Google Trends (Alphabet Inc), a publicly accessible website that analyzes the popularity of specific words or phrases entered into Google Search (Alphabet Inc), was used to obtain monthly search volumes for each term from January 2018 to December 2024 (n=84 monthly observations) [11,12]. Google Trends provides a normalized 0 to 100 relative search volume (RSV) scale rather than absolute search counts; 0 indicates a low RSV, and 100 represents the point of peak relative interest within the queried range. Google Trends does not offer absolute query counts, demographic detail, or the exact denominator population for any term [12-15]. These constraints are limitations of the platform rather than a deliberate study design, limiting our ability to report absolute counts alongside percentages that would be conventional for count-based epidemiologic data. We used specific search terms rather than Google Trends “Topics,” a related feature that groups multiple related queries and entities under a single label using an internal, undisclosed classification algorithm. Because a Topic’s exact query composition is not published by Google, its precise boundaries cannot be independently verified or reported. Specific search terms, by contrast, correspond to an exact, reproducible query string, which we prioritized for methodological transparency at the cost of a possibly narrower capture of related search phrasing; a parallel Topics-based analysis is a reasonable direction for future work.

### Data Processing and Cleaning

The monthly data were downloaded and imported using the programming language Python (version 3.9.23; Python Software Foundation). Data were checked for missing values and outliers; no observations required exclusion across the 6 primary terms.

### Segmented (Interrupted Time Series) Regression

To distinguish a pandemic-specific shift from a pre-existing linear trend, we specified a segmented regression model with 2 structural break points following the World Health Organization’s (WHO) official pandemic timeline: March 2020 (pandemic onset) and May 2023 (WHO Public Health Emergency of International Concern [PHEIC]) [16,17]:


$$RSV=\beta_{0}+\beta_{1}(t)+\beta_{2}(D_{1})+\beta_{3}(t_{1})+\beta_{4}(D_{2})+\beta_{5}(t_{2})+\varepsilon$$

where *β*_1_ estimates the prepandemic baseline trend. *β*_2_ and *β*_3_ estimate the level and slope change at pandemic onset. *β*_4_ and *β*_5_ estimate the level and slope change at postpandemic onset. *t* is the number of months elapsed since January 2018. *D*1 is a binary indicator for the pandemic or postpandemic period. *t*1 is the number of months elapsed since March 2020 (0 if before the pandemic). *D*2 indicates the postpandemic period as a binary variable. *t*2 is the number of months elapsed since May 2023 (0 for any month before). Newey-West (heteroskedasticity and autocorrelation consistent [HAC]) SEs were used to correct for residual autocorrelation, with a lag length of 3 months selected via the standard rule of thumb for 84 monthly observations, applied consistently across all primary and sensitivity term groups [18,19].

Each term was evaluated using a joint *F* test to test if the pandemic period and postpandemic period differed from the prior trend as a whole. This test assessed whether the pandemic period was statistically distinguishable from the pre-existing trajectory. *P* values for the pandemic effect and postpandemic effect tests were corrected across the 6 terms using both Bonferroni and Benjamini-Hochberg (false discovery rate [FDR]) procedures [20].

### Sensitivity Analysis

To assess whether the 6 primary terms analyzed for a pandemic-associated pattern reflected a term selection artifact rather than a broader signal, an identical segmented regression and joint test framework was applied to 2 additional sets of terms. The first set comprised 5 colloquial synonyms of the 6 primary terms: “excoriation disorder” (synonym of “dermatillomania”), “hair pulling” (synonym of “trichotillomania”), “compulsive skin picking” (synonym of “skin picking”), “stress eczema” (synonym of “eczema flare up”), and “itching anxiety” (related to “anxiety skin picking”). Another exploratory sensitivity set comprised 4 additional stress- or skin-adjacent terms that captured a broader range than direct synonyms: “acne,” “stress acne,” “stress hives,” and “hair loss from stress.” Both sets were subjected to Bonferroni and FDR corrections to allow a better comparison with the primary 6-term group.

## Results

### Overview

Table 1 summarizes the segmented regression coefficients and joint significance tests for the 6 primary search terms. Table 2 summarizes the synonymic term sensitivity analysis. Table 3 summarizes the secondary exploratory sensitivity analysis.

**Table 1.** Segmented regression results for the 6 primary search terms.

Terms	Baseline β_1_		Pandemic level β_2_		Pandemic slope Δβ_3_		Postpandemic level β_4_		Postpandemic slope Δβ_5_		Joint pandemic significance test		Joint postpandemic significance test	
	RSV points/mo	*P* value	RSV points/mo	*P* value	RSV points/mo	*P* value	RSV points/mo	*P* value	RSV points/mo	*P* value	Bonferroni *P* value	FDR^a^ *P* value	Bonferroni *P* value	FDR *P* value
Skin picking	0.305	.005	−0.089	.97	0.444	.008	−3.939	.30	−0.436	.03	.19	.04	.32	.08
Eczema flare-up	0.229	.08	−3.394	.42	0.562	.003	−0.626	.82	−0.078	.77	.08	.03	>.99	.93
Dermatillomania	0.063	.48	15.198	<.001	−0.116	.45	−4.753	.35	0.332	.23	.007	.007	>.99	.43
Anxiety skin picking	−0.164	.46	13.118	.02	0.695	.01	−21.645	<.001	−0.896	.007	.02	.01	<.001	.001
Trichotillomania	0.052	.60	5.111	.03	−0.040	.80	−7.990	.06	0.206	.15	.45	.08	.02	.006
Stress rash	1.487	.003	−29.409	.009	−1.196	.01	−1.315	.70	−0.613	<.001	.21	.04	.009	.005

a FDR: false discovery rate.

**Table 2.** Synonymic term sensitivity analysis: segmented regression joint tests paired with primary terms.

Terms (paired primary terms)	Pandemic period			Postpandemic period			Match?^a^
	*P* value (raw)	Bonferroni *P* value	FDR^b^ *P* value	*P* value (raw)	Bonferroni *P* value	FDR *P* value	
Excoriation disorder (dermatillomania)	.01	.06	.04	.046	.23	.08	Partial
Hair pulling (trichotillomania)	.02	.08	.04	.62	>.99	.78	No
Compulsive skin picking (skin picking)	.59	>.99	.74	<.001	.002	.002	No
Stress eczema (eczema flare-up)	.08	.41	.14	.92	>.99	.92	No
Itching anxiety (anxiety skin picking)	.86	>.99	.86	.002	.009	.004	Partial

a The “Match?” column indicates whether each synonym term reached the same statistical conclusion (significant vs not significant) as its paired primary term, with “Partial” meaning the two agreed on only one of the two tested effects (or agreed in direction but not in significance under both corrections), and “No” meaning they reached opposite conclusions.

b FDR: false discovery rate.

**Table 3.** Secondary exploratory sensitivity analysis: segmented regression joint tests for 4 additional terms.

Terms	Joint pandemic effect			Joint postpandemic effect		
	*P* value (raw)	Bonferroni *P* value	FDR^a^ *P* value (BH^b^)	*P* value (raw)	Bonferroni *P* value	FDR *P* value (BH)
Acne	.02	.09	.07	<.001	<.001	<.001
Stress acne	.16	.64	.21	.007	.03	.009
Stress hives	.04	.15	.07	.36	>.99	.36
Hair loss from stress	.72	>.99	.72	<.001	<.001	<.001

a FDR: false discovery rate.

b BH: Benjamini-Hochberg.

### Skin Picking

“Skin picking” showed a significant upward prepandemic baseline trend (*β*_1_=+0.31 RSV points/month; *P*=.005). The slope increased further during the pandemic period (*β*_3_=+0.44 RSV points/month; *P*=.008), and the joint pandemic effect test reached significance under FDR correction (*P*=.04) but not under the stricter Bonferroni correction (*P*=.19). The postpandemic joint test was not significant under either correction (Bonferroni *P*=.32; FDR *P*=.08).

### Eczema Flare-Up

“Eczema flare up” searches also trended upward across the study period, although the prepandemic baseline slope was only marginally significant (*β*_1_=+0.23 RSV points/month; *P*=.08). The slope increased further at pandemic onset (*β*_3_=+0.56 RSV points/month; *P*=.003), and the joint pandemic effect test reached significance under FDR correction (*P*=.03) but not under the Bonferroni correction (*P*=.08). This was the same pattern observed for “skin picking.” The postpandemic joint test was not significant under either correction (*P*=.93).

### Dermatillomania

“Dermatillomania” showed no significant prepandemic baseline trend (*β*_1_=+0.06 RSV points/month; *P*=.48), but a large, statistically significant level shift at pandemic onset (*β*_2_=+15.20 RSV points; *P*<.001). The joint pandemic effect test was significant and survived both Bonferroni (*P*=.007) and FDR (*P*=.007) corrections. This finding was significant under the stricter Bonferroni correction. No significant postpandemic reversal was detected (*P*=.36).

### Anxiety Skin Picking

“Anxiety skin picking” showed a significant level shift (*β*_2_=+13.12 RSV points; *P*=.02) and slope increase (*β*_3_=+0.70 RSV points/month; *P*=.01) at pandemic onset, and the joint pandemic effect test survived both Bonferroni (*P*=.02) and FDR (*P*=.01) corrections. This term also showed the clearest reversal in the dataset: a significant level drop (*β*_4_=−21.64 RSV points; *P*<.001) and slope reversal (*β*_5_=−0.90 RSV points/month; *P*=.007) at postpandemic onset, with the joint postpandemic effect test remaining highly significant after correction (both *P*<.001). “Anxiety skin picking” showed both a pandemic onset rise that was significant under Bonferroni correction and a significant postpandemic reversal.

### Trichotillomania

“Trichotillomania” showed no significant prepandemic trend (*P*=.60) and no significant pandemic onset effect. The joint pandemic effect test did not reach significance under either correction (Bonferroni *P*=.45; FDR *P*=.08). A significant postpandemic joint effect was detected instead (joint test Bonferroni *P*=.02; FDR *P*=.006), although neither the level shift (*β*_4_=−7.99 points; *P*=.06) nor the slope change (*β*_5_=+0.21 RSV/points; *P*=.15) was individually significant—the reversal was detectable only when both were considered jointly.

### Stress Rash

“Stress rash” exhibited a steep and statistically significant prepandemic baseline rise (*β*_1_=+1.49 RSV points/month; *P*=.003)—largely driven by a sharp increase in 2019, prior to the pandemic. At pandemic onset, both the level (*β*_2_=−29.41 RSV points; *P*=.009) and slope (*β*_3_=−1.20 RSV points/month; *P*=.01) dropped significantly, and the joint pandemic effect test reached significance under FDR correction (*P*=.04) but not under the Bonferroni correction (*P*=.21). A further significant slope reversal occurred in the postpandemic period (*β*_5_=−0.61 RSV points/month; *P*<.001; joint postpandemic effect test significant under both corrections; Bonferroni *P*=.009). Formally modeling this term showed that its prepandemic trajectory was already elevated and that the pandemic period corresponded to a decline rather than an increase. This direction differed from those observed for dermatillomania and anxiety skin picking, and the pandemic-onset finding depended on the correction threshold applied.

### Sensitivity Analysis Results: Synonymic Terms

“Excoriation disorder,” the clinical synonym for “dermatillomania,” showed a pandemic onset effect significant under FDR correction (*P*=.04) but not under the Bonferroni correction (*P*=.06), suggesting only partial agreement with “dermatillomania’s” Bonferroni-robust finding. “Hair pulling,” the colloquial synonym for “trichotillomania,” showed a pandemic onset effect significant under FDR correction (*P*=.04) but not under the Bonferroni correction (*P*=.08); “trichotillomania” itself showed no significant effect under either correction, so this pair showed divergent findings. “Compulsive skin picking” showed no significant pandemic onset effect (*P*=.59), while its primary term, “skin picking,” reached FDR significance (*P*=.04). This pair also showed divergent findings. “Stress eczema” likewise showed no significant effect (FDR *P*=.14), while “eczema flare up” reached FDR significance (*P*=.03), indicating another divergence. “Itching anxiety” showed no significant pandemic onset effect (*P*=.86) and therefore did not replicate the Bonferroni-robust effect found for “anxiety skin picking”; it did, however, replicate the postpandemic reversal (Bonferroni *P*=.009; FDR *P*=.004). Overall, only 1 of 5 synonym pairs (“dermatillomania”–“excoriation disorder”) showed even partial agreement for the pandemic onset finding.

### Sensitivity Analysis Results: Exploratory Terms

None of the 4 secondary exploratory terms showed a pandemic onset effect surviving either correction: “acne” (Bonferroni *P*=.09; FDR *P*=.07) and “stress hives” (Bonferroni *P*=.15; FDR *P*=.07) came closest but did not reach significance under FDR correction; “stress acne” (FDR *P*=.21) and “hair loss from stress” (FDR *P*=.72) showed no evidence of a pandemic onset effect. Postpandemic reversals, by contrast, were common and robust: “acne,” “stress acne,” and “hair loss from stress” all showed significant postpandemic shifts surviving both corrections (all Bonferroni *P*≤.03), while “stress hives” did not (*P*=.36).

## Discussion

### Principal Findings

This study identified statistically significant pandemic-associated changes in public search interest in dermatillomania and anxiety skin picking, whereas evidence for other search terms was weaker and more sensitive to our analytic approach. Sensitivity analyses demonstrated that several findings depended on search term selection, highlighting the importance of caution when interpreting Google Trends data.

Psychodermatology bridges the fields of psychiatry and dermatology, focusing on the complex interaction between skin health and psychological states. The American Psychiatric Association suggests that more than one-third of dermatological patients have psychological concerns [21]. The pathophysiological connection between dermatological and psychological conditions is multifactorial, involving the hypothalamic-pituitary-adrenal (HPA) axis, neuroimmune signaling, and peripheral nerve activation [22]. Psychological stress triggered by external events can alter immune function, increase proinflammatory cytokines, or disrupt the skin barrier, exacerbating conditions such as acne, eczema, psoriasis, and pruritus [3,23]. These intertwined pathways emphasize a need for treatment that addresses both dermatologic and mental health concerns in patients.

### Skin Picking Behaviors

Skin picking behaviors were encompassed by the search terms “skin picking,” “dermatillomania,” and “anxiety skin picking.” “Skin picking” search interest showed a significant pandemic onset increase in RSV (+0.444 slope change) under the FDR correction but not under the Bonferroni correction. “Dermatillomania” and “anxiety skin picking” showed the strongest evidence among the 6 terms for a significant pandemic onset effect under both the FDR and Bonferroni corrections. “Dermatillomania” experienced a large jump in RSV at pandemic onset (+15.198), while “anxiety skin picking” showed an increase in the RSV slope (+0.695) and a large jump in RSV at pandemic onset (+13.118). “Anxiety skin picking” also had a significant postpandemic reversal in the RSV slope (–0.896) and a large decrease in the estimated level of RSV (–21.645). It could be that “dermatillomania” captures a more clinical lexicon that received increased interest during the pandemic, and “anxiety skin picking” provides more specific language than “skin picking” alone. Because “skin picking” is broad, a more stable base rate of general searches is more difficult to distinguish from its prepandemic trend. Skin picking disorder has documented associations with anxiety, depression, and panic disorder [24], conditions widely reported to have increased during the pandemic [25]; this could be related to the pandemic onset of an increase in RSV, but this is purely speculative. Visual inspection of the raw “skin picking” search data (Figure 1 [16,17]) suggests a recurring decline each December; this is a descriptive observation from the unadjusted data rather than a formally tested seasonal effect (Table 1).

**Figure 1.**
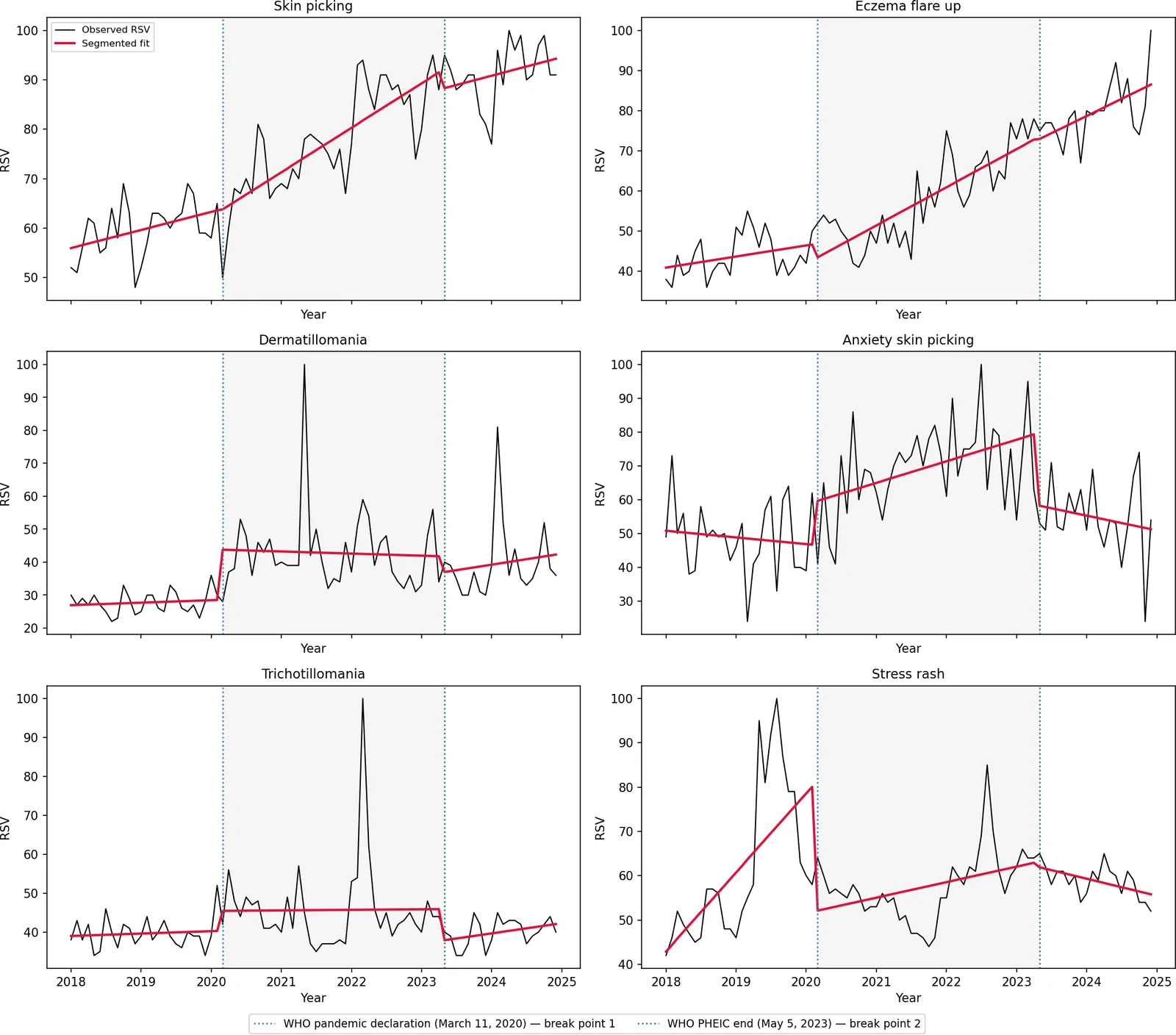
Segmented regression fits overlaid on observed monthly relative search volume (RSV) for the 6 primary search terms from January 2018 to December 2024. The shaded region indicates the pandemic period. The blue dotted line marks the World Health Organization (WHO) pandemic declaration on March 11, 2020 [16], corresponding to the model’s first break point; the green dotted line marks the end of the WHO Public Health Emergency of International Concern (PHEIC) on May 5, 2023 [17], corresponding to the model’s second break point.

“Skin picking” search interest showed a significant pandemic onset increase in RSV (+0.444 slope change) under the FDR correction but not under the Bonferroni correction. “Dermatillomania” and “anxiety skin picking” showed the strongest evidence among the 6 terms for a significant pandemic onset effect under both the FDR and Bonferroni corrections. “Dermatillomania” experienced a large jump in RSV at pandemic onset (+15.198), while “anxiety skin picking” showed an increase in the RSV slope (+0.695) and a large jump in RSV at pandemic onset (+13.118). “Anxiety skin picking” also had a significant postpandemic reversal in the RSV slope (–0.896) and a large decrease in the estimated level of RSV (–21.645). It could be that “dermatillomania” captures a more clinical lexicon that received increased interest during the pandemic, and “anxiety skin picking” provides more specific language than “skin picking” alone. Because “skin picking” is broad, a more stable base rate of general searches is more difficult to distinguish from its prepandemic trend. Skin picking disorder has documented associations with anxiety, depression, and panic disorder [24], conditions widely reported to have increased during the pandemic [25]; this could be related to the pandemic onset of an increase in RSV, but this is purely speculative. Visual inspection of the raw “skin picking” search data (Figure 1 [16,17]) suggests a recurring decline each December; this is a descriptive observation from the unadjusted data rather than a formally tested seasonal effect (Table 1).

### Hair-Related Stress Responses

Hair-related stress responses were encompassed by the search term “trichotillomania.” “Trichotillomania” experienced no significant pandemic onset effect under either correction. The joint postpandemic test was significant under both corrections (Bonferroni *P*=.02; FDR *P*=.006), although neither the level shift (*β*_4_=−7.99 RSV points; *P*=.06) nor the slope change (*β*_5_=+0.21 RSV points/month; *P*=.15) was individually significant. The reversal was detectable only when both were considered jointly. Body-focused repetitive behaviors increased during the pandemic [26,27], but this dataset does not provide adequate support for the corresponding pandemic onset shift. RSV for “trichotillomania” alone is not a reliable proxy for the symptom timing (Table 1).

### Stress-Influenced Inflammatory Dermatoses

Stress-influenced inflammatory dermatoses were encompassed by the search term “stress rash.” “Stress rash” RSV showed a significant decrease in the RSV (–1.196) and estimated RSV level (–29.409) at the onset of the pandemic under the FDR correction, reversing its sharp increase in the preceding year, 2019 (Figure 1 [16,17]). The postpandemic era was associated with a further significant decrease under both the FDR and Bonferroni corrections (–0.613; Table 1). One possible explanation is that during the early pandemic, public attention shifted toward COVID-19–related symptoms and information seeking, which may have reduced searches for the specific term “stress rash.” Alternatively, the decline may reflect changes in search terminology rather than a true reduction in stress-related dermatologic symptoms. Because Google Trends cannot determine users’ motivations, these explanations remain speculative.

### Stress-Influenced Chronic Eczematous Dermatoses

Stress-influenced chronic eczematous dermatoses were encompassed by the search term “eczema flare up.” A significant increase in the RSV slope was found for “eczema flare up” under the FDR correction but not under the Bonferroni correction (+0.562). Frequent handwashing and psychological stress during the pandemic have been proposed as potential contributors to eczema symptom exacerbation [28,29]. Although the FDR correction found significance, the increase in RSV for “eczema flare up” could have followed the upward trend from the prepandemic era (Figure 1 [16,17]). “Eczema flare up” did not experience any significant change in the RSV slope in the postpandemic era (Table 1).

### Sensitivity Analysis

A sensitivity analysis for synonyms of the original 6 terms was chosen to test if the primary findings depended on the specific phrasing chosen for each term. Only 1 of 5 synonym pairs (“dermatillomania”–“excoriation disorder”) showed partial agreement with its matched primary term for the pandemic onset effect, with significance observed only under the FDR correction. The remaining 4 pairs showed divergent findings for the pandemic onset effect: “hair pulling” showed an FDR-level effect despite “trichotillomania” showing none; “compulsive skin picking” and “stress eczema” showed no effect despite their primary terms (“skin picking” and “eczema flare up”) reaching FDR significance; and “itching anxiety” showed no onset effect despite “anxiety skin picking’s” Bonferroni finding, although the 2 did agree on a postpandemic reversal. This sensitivity analysis argues against treating the primary pandemic onset findings as robust to search term phrasing and should be reported as a limitation rather than a validation of those findings (Table 2).

The secondary exploratory analysis used general terms to test the robustness of the primary findings. The 4 terms, “acne,” “stress acne,” “stress hives,” and “hair loss from stress,” showed no significant pandemic onset effect under either correction. “Acne,” “stress acne,” and “hair loss from stress” showed significant postpandemic increases (Figure 2 [16,17]). Taken together, the 2 sensitivity analyses provide less support for the robustness of the findings for the primary 6 terms. The strongest and most consistent findings remain the pandemic onset effects that survived Bonferroni correction for “dermatillomania” and “anxiety skin picking,” and the postpandemic reversals for “anxiety skin picking,” “trichotillomania,” and “stress rash.”

**Figure 2.**
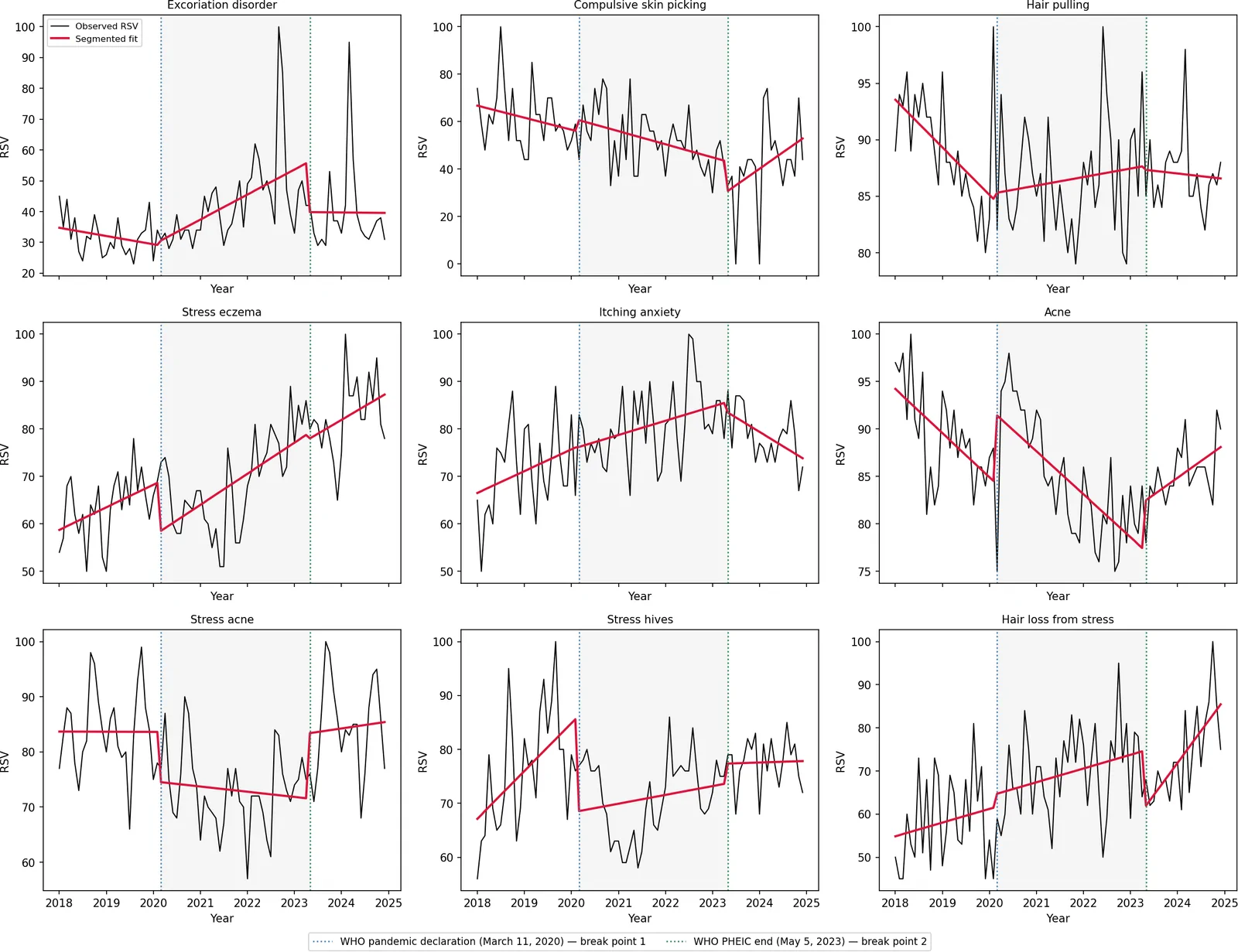
Segmented regression fits overlaid on observed monthly relative search volume (RSV) for the 9 sensitivity terms (synonymic terms and 4 secondary exploratory terms), January 2018 to December 2024. The shaded region indicates the pandemic period. The blue dotted line marks the World Health Organization (WHO) pandemic declaration on March 11, 2020 [16], corresponding to the model’s first break point; the green dotted line marks the end of the WHO Public Health Emergency of International Concern (PHEIC) on May 5, 2023 [17], corresponding to the model’s second break point as in Figure 1 [16,17].

### Strengths and Limitations

The strengths of this study are the use of a segmented regression framework, anchored by the WHO’s official pandemic timeline, that tests for pandemic-associated shifts in RSV. Newey-West SEs address residual autocorrelation, and multiple-comparison corrections were applied across the 6 primary terms. Bonferroni and FDR corrections identify the findings as sensitive to the choice of correction. The sensitivity analyses provide a more comprehensive assessment of the 6 terms to determine whether the findings were affected by specific term selection or wording. The transparency of the sensitivity analysis shows that the primary findings may be inconsistent, but this is itself a strength of reporting.

The limitations are consistent with Google Trends analyses, as the RSV metric does not provide absolute search counts and does not disclose demographic information. Furthermore, it cannot be concluded who is searching for a specific term or why. Although Google accounts for an estimated 87% of internet searches in North America [30], there are other internet search engines that are excluded from the analysis. Internet access and search behavior may vary by region, income, and other potential factors that are not measurable within this dataset. Search interest is a proxy for public attention and cannot be used to infer disease incidence, prevalence, or diagnosis. The segmented regression does not include explicit seasonal (month-of-year) controls; seasonal patterns noted in the Discussion (eg, a December decline in “skin picking” searches) are descriptive observations from the raw data rather than formally tested effects and should be interpreted accordingly. Most importantly, the pandemic onset findings for 4 of the 6 primary terms (“skin picking,” “eczema flare up,” “trichotillomania,” and “stress rash”) did not hold up consistently across correction methods or sensitivity analyses. Only “dermatillomania” and “anxiety skin picking” showed pandemic onset effects that were robust across the correction methods and sensitivity analyses, along with the postpandemic reversals for “anxiety skin picking,” “trichotillomania,” and “stress rash.”

### Conclusions

We applied a segmented regression model, which adhered to the WHO’s official pandemic timeline, to Google Trends data from January 2018 through December 2024. This study finds that public search interest in “dermatillomania” and “anxiety skin picking” showed pandemic onset changes that were robust to multiple corrections. Evidence for “skin picking,” “eczema flare up,” and “stress rash” was present only under the FDR correction, and “trichotillomania” showed no pandemic onset effect. Postpandemic reversals were detected for “anxiety skin picking,” “trichotillomania,” and “stress rash.” Sensitivity analyses using synonymic and conceptually related search terms did not consistently reproduce the primary findings, indicating that most of these results are sensitive to search term phrasing and the choice of statistical correction. The findings support continued attention to the intersection of dermatology and mental health and illustrate that formal time series methods are necessary but may not always be sufficient on their own.

## Abbreviations

FDR: false discovery rate
HAC: heteroskedasticity and autocorrelation consistent
HPA: hypothalamic-pituitary-adrenal
PHEIC: Public Health Emergency of International Concern
RSV: relative search volume
WHO: World Health Organization
